# Conjunctival Ultraviolet Autofluorescence: A Systematic Review of Factors Affecting Observed Ocular Damage

**DOI:** 10.1007/s44402-026-00043-1

**Published:** 2026-02-27

**Authors:** Ishwarya Suresh Kumar, Katie Edwards, Katrina L. Schmid

**Affiliations:** Centre for Vision and Eye Research, Optometry and Vision Science, Faculty of Health, Queensland University of Technology, Brisbane, Queensland Australia

**Keywords:** Conjunctival ultraviolet autofluorescence, Myopia, Ocular sun exposure, Pterygium, Ultraviolet damage

## Abstract

**Purpose:**

Conjunctival ultraviolet autofluorescence (CUVAF) is a marker for ocular sun damage. While its potential is recognised in research, factors influencing its development and temporal dynamics remain poorly documented. This systematic review evaluates whether the size of CUVAF reflects recent ultraviolet (UV) exposure, lifetime cumulative damage or both.

**Recent findings:**

Google Scholar, PubMed, Embase, Scopus and ProQuest Global databases were searched for papers using defined search terms. Ninety-one articles were assessed and 35 studies using CUVAF as an indicator of sun exposure were analysed. Extracted data included study location, CUVAF area (mean/median, mm²)/presence or pattern, participant’s age, skin type, occupation, time spent outdoors, UV protection behaviour, the presence of UV eye disease and seasonal variations if assessed. Study quality was evaluated using the Newcastle–Ottawa-Scale. A locally weighted scatterplot smoothing (LOWESS) and polynomial curve were generated to visualise age-related trends. CUVAF area peaked in young adults and followed nonlinear, oscillating pattern with age. High ambient UV regions like Australia showed greater weighted average CUVAF (28.8 mm², range: 0.6–45.4 mm^2^) compared to moderate or lower UV regions such as India (6.8 mm², range: 4.3–11.1 mm^2^), the USA (7.5 mm², range: 5.5–9.2 mm^2^) and Europe (5.3 mm², range 0.4–6.4 mm^2^). Outdoor work and fair pigmentation were associated with larger CUVAF area. CUVAF size did not vary with season. Myopes had smaller average CUVAF areas than non-myopes (14.5 vs. 21.5 mm²). Eyes with UV-related eye disease exhibited larger average areas (43.4 vs. 20.7 mm^2^) compared to healthy eyes. There was no reported correlation between sunglass use and CUVAF. Artificial UV irradiation temporarily increased CUVAF area.

**Summary:**

Age-related oscillations in CUVAF, larger values in high-UV regions and transient increases following artificial UV exposure suggest that CUVAF may reflect both acute conjunctival responses and cumulative UV exposure. However, its ability to capture dynamic changes under natural environmental UV remains uncertain without longitudinal data.

Key Points

- Conjunctival ultraviolet autofluorescence demonstrates geographical variations, consistent with ambient ultraviolet levels.
- Larger conjunctival ultraviolet autofluorescence area is linked to either long-term ultraviolet exposure (over several months or years) or short-term high ultraviolet exposure.
- Conjunctival ultraviolet autofluorescence may indicate either recent and/or cumulative cellular damage, but supporting evidence is limited.

## Introduction

The ocular surface is directly exposed to sunlight [1] and prolonged ultraviolet (UV) radiation increases the risk of UV-related ocular diseases, including pterygium, cataract, eyelid malignancies and ocular surface squamous neoplasia (OSSN) [2]. UV absorption induces cellular damage and alters a range of cell components, including collagen, elastin, lysosomes, mitochondria, cytokines, nicotinamide adenine dinucleotide, tryptophan, lipofuscin and matrix metalloproteinases (MMPs) [3, 4]. These altered intracellular contents may result in autofluorescence emission from affected tissues [4].

Autofluorescence occurs when fluorophores absorb short-wavelength light and emit longer wavelengths, a principle used by the Wood’s lamp, originally developed for dermatological applications, to detect abnormal accumulations of endogenous chromophores and pigmentation in the skin [5]. Ooi et al. [6] described ocular surface UV autofluorescence and developed a non-invasive technique to quantify autofluorescence area (mm²) and intensity (brightness signal in pixels × 10^−^³) in the nasal and temporal bulbar conjunctiva [3], reporting associations with pterygium, correlations with ocular sun exposure and proposing conjunctival ultraviolet autofluorescence (CUVAF) as an objective marker of UV-induced ocular damage [6, 7].

Historically, sun exposure has been assessed using diaries, surveys, dosimeters and sensors [8], but self-reported methods are often inaccurate and subject to recall bias [9, 10], whereas personal UV dosimeters (e.g., polysulphone-film badges) provide objective daily exposure data [11] and wearable UV sensors enable longer monitoring periods [12]. UV exposure causes photodamage leading to photoaging and skin cancer, which can be detected using non-invasive imaging such as skin surface topography [13] and optical coherence tomography (OCT) [14]. In the ocular context, CUVAF is regarded as a reliable, non-invasive biomarker of subclinical UV damage [6].

CUVAF’s utility as an indicator of UV exposure is supported by its anatomical distribution. Nasal CUVAF is typically more pronounced than temporal CUVAF due to the peripheral light focusing effect [6, 15–19], although not all studies report this asymmetry [20, 21]. Anatomical features may also influence ocular UV exposure. Variations in orbital depth, brow ridge prominence and overall facial morphology can affect the amount of UV radiation reaching the eye [22]. A mannequin-based study [23] demonstrated that differences in facial structure, particularly the superciliary arch and glabella, can reduce conjunctival UV exposure, suggesting that individuals with deeper-set eyes or more prominent foreheads may experience less UV-induced damage. Therefore, such anatomical variability may contribute to individual differences in CUVAF area.

Outdoor activity is another key determinant of CUVAF. Individuals who spend more time outdoors tend to exhibit larger areas of CUVAF [24–28]. Importantly, CUVAF can also be seen in eyes with clinically normal ocular surfaces, indicating its potential as a marker of early UV-induced damage [15]. Eyes with ophthalmohelioses typically show extensive CUVAF due to UV-injured cells [29, 30]. CUVAF has also been used in myopia research to quantify outdoor exposure, with studies reporting an inverse association between CUVAF area and myopia [31].

Although CUVAF is a promising marker of UV exposure, several aspects remain unclear. These include its temporal dynamics, factors influencing its size and intensity and whether behaviour changes, such as sun protection, can alter its development or progression [20]. Moreover, ocular sun exposure and CUVAF development are influenced by multiple confounding factors, including age [32], outdoor activity [15], use of ocular protection [32], geographical location and UV-related eye conditions such as pinguecula [33], pterygium [7] or OSSN [30].

This review aims to consolidate current evidence on CUVAF to determine whether the fluorescent area reflects lifetime cumulative damage, recent cumulative damage (over several months or years) or a combination of past and ongoing cellular damage. It will also examine how factors such as age, ethnicity, behavioural patterns and seasonal variation influence CUVAF size, thereby enhancing the understanding of its development, dynamics and clinical relevance.

## Methodology

Published studies examining the appearance and clinical relevance of CUVAF were reviewed systematically. These investigations explored its associations with geography, age, skin pigmentation, seasonal variations, time spent outdoors, the impact of ocular protection, UV exposure pattern variations with refractive error, its utility as a risk indicator for ophthalmohelioses, longitudinal changes and the effects of artificial UV exposure. The review was conducted in accordance with the Preferred Reporting Items for Systematic Reviews and Meta-Analyses (PRISMA) guidelines [34] and was registered with the International Prospective Register of Systematic Reviews (PROSPERO CRD: 42024498587)

### Search strategies

**Fig. 1:**
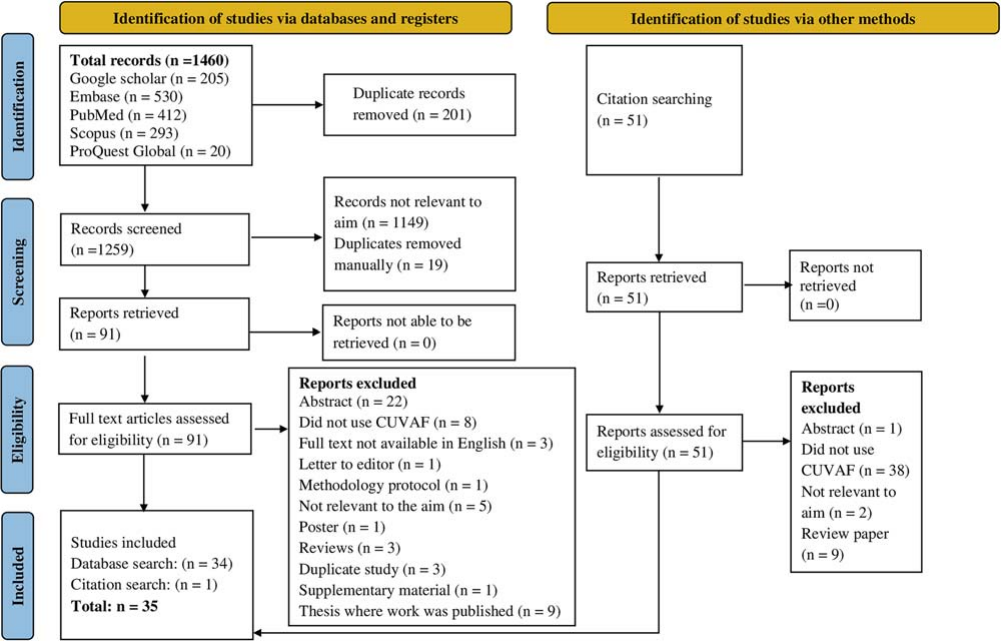
Flow diagram of the database search and article selection process. CUVAF conjunctival ultraviolet autofluorescence.

Searches were conducted using the following databases, Google Scholar, PubMed, Embase, Scopus and ProQuest Global. The term “conjunctival ultraviolet autofluorescence” was used as the initial scoping search term in PubMed to identify key literature on the topic and assess the volume of existing research, which resulted in 38 papers. However, as no MeSH term was available for CUVAF, a more extensive search with following key terms, (1) “conjunctival ultraviolet autofluorescence” OR “CUVAF” OR “UVAF”, (2) “objective marker of ocular sun exposure”, (3) “ocular surface disease”, (4)“pterygium” or “pterygia”, (5) “pinguecula”, (6)“ocular surface squamous neoplasia” or “OSSN” (7) OR “myopia” was conducted, this ensured no relevant articles were missed. Searches were conducted and updated between December 2023 and February 2025. The summary of the search protocol is provided in Fig.1.

### Study inclusion and exclusion criteria

All observational studies (cross-sectional, case-control and cohort studies), case series and longitudinal studies published in English were included. Studies in other languages were also included if the English translation was available (*n* = 2) or could be understood using Google Translate (*n* = 0). Studies reporting the association of CUVAF with UV exposure were selected without any year limitation and all relevant published studies were assessed.

Studies were excluded based on the following criteria: (1) duplicate publications using the same CUVAF data (the study reporting the largest number of participants and/or studies reporting different confounding factors and/or different measures of interest were selected; theses were excluded if the work had been published subsequently); (2) studies involving animal models or in vitro conditions; (3) articles that did not report primary data, such as reviews and (4) studies in which CUVAF was not quantified, for example those using techniques like cell staining.

### Study selection

The primary source articles were imported into Covidence software (Veritas Health Innovation, covidence.org), which automatically removed duplicates and facilitated title and abstract screening (ISK). Full-text reviews were conducted for eligible studies, and any discrepancies were resolved by discussion among reviewers (ISK, KE, and KLS). Reference lists and citations of included studies were examined to identify additional relevant publications. In total, 35 studies were included (Fig. 1).

### Data extraction

From the studies selected for full-text review, data were extracted and entered in Microsoft Excel (Microsoft.com) (ISK) and data entry checking was conducted subsequently (KE, KLS). Extracted variables included: authors, publication year, study title, country of origin, sample size, participant gender, age range, ocular condition, time spent outdoors, CUVAF-related findings, statistical analysis performed and reported study limitations.

### Risk of bias assessment

The Newcastle–Ottawa Scale (NOS) was used to assess potential risk of bias in each study. This scale uses a star rating system, with a maximum of nine stars based on three broad categories: selection, comparability and exposure/outcome assessment [35].

### Quantitative analysis

In the graphs, CUVAF values are presented as weighted averages based on each study’s population (e.g., myopic vs. non-myopic; CUVAF present vs. absent). For studies that reported CUVAF areas for participants with and without ophthalmohelioses, the values from participants with UV-associated ocular diseases were excluded. The values are reported as either means or medians, consistent with the original values presented in the respective studies. Most studies reported CUVAF area as the median due to the skewed distribution of the data [27]. However, a few studies presented mean values, stating the distribution was symmetrical. This suggests that in such cases, the estimated median is likely to approximate the reported mean [36].

Three studies [15, 19, 37] provided data for the right eye only. To estimate the overall total CUVAF for both eyes, a correction factor was applied, using the assumption of bilateral symmetry, [19] whereby the right eye measurement was simply doubled. In four studies [15, 17, 19, 37], zero values were excluded from the calculation of the average CUVAF area. Therefore, a weighted mean formula was used to estimate the overall CUVAF (corrected data were used in graphs). The data used in the graphs were cross-sectional in nature and the weighted mean and median CUVAF values should be considered as descriptive summaries rather than precise normative estimates, as methodological and population heterogeneity limit the precision of cross-study comparisons.

The relationship between age and CUVAF distribution was examined using scatter plots with locally weighted scatterplot smoothing (LOWESS) and confirmatory spline regression models, using GraphPad prism (Version 10.4.1 for Windows, graphpad.com). LOWESS is a nonparametric method that does not assume any specific functional form (e.g., linear) allowing it to model complex, non-linear data trends flexibly [38]. Polynomial and LOWESS smoothing are useful for visualising complex patterns in the non-linear age-related data, but do not allow for hypothesis testing. The generalised additive model (GAM) is preferred for formal modelling and statistical analysis; however, it requires individual-level raw data of each participant or simulated individual data, i.e., aggregated data cannot be used.

## Results

### Study characteristics

Following database searches and full-text assessments (Fig. 1), 35 articles met the inclusion criteria. Of these, 17 studies were conducted in Australia (Table 1), nine in Europe (Table 2), five in India (Table 3) and two each in the USA and other regions (Table 4). Most studies (*n* = 28) reported quantitative measurements of CUVAF area (mm^2^), using medians (*n* = 14), means (*n* = 10) or both (*n* = 4). Seven studies reported only qualitative assessments, such as the presence, absence or pattern of the autofluorescence and two European studies described both CUVAF area and intensity (Tables 1- 4).

**Table 1:** Conjunctival ultraviolet autofluorescence (CUVAF) investigations in Australia.

Author/Year	Study design	Location	Age (yr)	Population	Number of subjects (*n*)	Mean/Median CUVAF area (mm^2^)	Outdoor exposure time (h)	CUVAF analysis methods	Reported findings
Ooi et al., 2006 [6]	Cross-sectional	Australia/Sydney	3–5	Without CUVAF	48	Nasal CUVAF was seen in 36 eyes and temporal in 32 eyes	NA	Adobe Photoshop CS4 Extend using standard protocol	The prevalence of fluorescent regions increased with age.
				With CUVAF	23				
Ooi et al., 2007 [7]	Case series	Australia/Sydney	26–62	Pterygium	14	CUVAF was more commonly seen at the limbus and leading edge of pterygium.	NA	NA	CUVAF represents active cellular damage within the pterygium.
Sherwin et al., 2011 [27]	Cross-sectional	Australia^¢^/Norfolk Island	15–70+	Distribution of CUVAF in NIES	641	Median: 28.1 (IQR: 14.5–48.2) Mean: 33.6 Range: (0–201)	NA	Manual tracing with imaging software	There was an inverse relationship with age and CUVAF area. Males employed in outdoor work had higher CUVAF area than females.
Sherwin et al., 2012 [43]	Cross-sectional	Australia^¢^/Norfolk Island	16–85	Reliability and validity of CUVAF	49	Indoor-median: 23.2 (IQR: 9.7–35.2)	NA	Adobe Photoshop CS4 Extend using standard protocol	Increasing time outdoors was strongly linked to larger total CUVAF area in summer and winter.
						Outdoor-median: 33.9 (IQR: 21.6– 58.1)			
Sherwin et al., 2012 [28]	Cross-sectional	Australia^¢^/Norfolk Island	15–89	Non-myopes	498	Median: 28.6	NA	Manual delineation followed by Adobe Photoshop CS4 Extended	There was a dose–response relationship, with increasing quartile of CUVAF being associated with reduced odds of myopia.
				Myopes G1 SE ≤ − 1.0 D G2 SE ≤ − 0.5D	G1: 47 G2: 91	Median G1: 16.6 G2: 24.5			
Sherwin et al., 2013 [44]	Cross-sectional	Australia^¢^/Norfolk Island	15–89	Without pterygium	571	Median: 26 (IQR 13.7–46.2)	NA	Adobe Photoshop CS4 Extend using standard protocol	Individuals with larger CUVAF area had increased risk of pterygium. Dose–response relationship was seen with increasing quartile of CUVAF and odds of pterygium.
				With pterygium	70	Median: 43.4 (IQR 25.5–61.9)			
McKnight et al., 2014 [26]	Population-based cross-sectional	Australia^¥^/Perth	19–22	Non-myopes	1017	Median: 47.9 (IQR 23.2–72.5)	NA	Adobe Photoshop CS4 Extend using standard protocol	Inverse relationship between myopia and CUVAF area and thus ocular sun exposure.
				Myopes SE - <0.50	311	Median: 31.9 (IQR 10.6–55.9)			
McKnight et al., 2015 [16]	Cross-sectional population-based	Australia^¥^/Perth	19–22	Without pterygium	1328	Median: 44 (IQR 20.2–69)	NA	Adobe Photoshop CS4 Extend using standard protocol	CUVAF was larger on the nasal than temporal conjunctiva. CUVAF was associated with increased time spent outdoors and increased prevalence of pterygium.
				With Pterygium	16	Median: 73.4 (IQR 48.3–94.7)			
Yazar et al., 2015 [45]	Longitudinal	Australia/Tasmania, Brisbane and Perth	TEST: 5-51 BATS: 13–28 Raine: 18-22	NA	1251	Median:TEST: 28.7 (IQR 15-42.3) BATS:45.4 (IQR 26.8–68.5) Raine:44.2 (IQR 20.3 –69.8)	NA	Adobe Photoshop CS4 Extend using standard protocol	Individuals living closer to equator had larger CUVAF areas compared with individuals living away from equator (45.5 vs. 28.7). Suggested that genes (e.g., snip rs1060043) were associated with CUVAF.
Sun et al., 2017 [17]	Cross-sectional	Australia/Melbourne	5–15	Without CUVAF	286	0	Average:<3 h/day Average: ≥3 h/day	Adobe Photoshop CS4 Extend using standard protocol	CUVAF was detected in children over 8 years. The odds of CUVAF increased with increasing age and fair pigmentation. Suggested CUVAF was an objective marker of past sun exposure.
				With CUVAF	53	Median: 3.8 (IQR: 1.5–2.9)			
Charng et al., 2019 [46]	Cross-sectional	Australia^¥^/Perth	Gen1: 40–82	Non-myopes vs. Myopes SE ≤−0.50D	561	Mean: 29.8 ± 26.4 vs. 27.7 ± 27.8	NA	Custom made semi-automated MATLAB analysis tool	CUVAF area was larger in non-myopes than myopes.
			Gen2: 20–28	Non-myopes vsMyopes SE ≤−0.50D	316	Mean: 46.8 ± 31.6 vs. 42.7 ± 29.4			
Lingham et al., 2021 [68]	Non-randomised controlled	Australia/Perth	25–30	Non-myopes	214	Larger CUVAF area was seen with greater outdoor times	NA	Custom made semi-automated MATLAB^R^ analysis software	Spending more time outdoors in both childhood and adolescence was associated with larger CUVAF area.
				Myopes SE ≤ −0.50D	89				
Stevenson et al., 2021 [47]	Cross-sectional	Australia^¥^/Perth	40–82	Without pterygium	911	Median: 20.7 (IQR: 30.7)	25 ± 18.1 h/week	Custom made semi-automated MATLAB^R^ 2011b	CUVAF was larger in eyes with pterygium than without. Higher CUVAF area, male sex and outdoor occupation were associated with an increased risk of pterygium
				With pterygium	59	Median: 37 (IQR: 33.6)	26.6 ± 18.3 h/week		
Lee et al., 2022 [48]	Longitudinal	Australia^¥^/Perth	20–28	Non-myopes	444	Median: 42.8 (IQR 23.3–65.4)	Mean: 19.1 ± 21.1 h/week	NA	Incident myopia was associated with smaller CUVAF area and less sun exposure.
				Myopes SE < −0.50D	72	Median: 34 (IQR 14.8–52.7)	Mean: 12.6 ± 12.4 h/week		
Rajasingam et al., 2023 [37]	Cross-sectional	Australia/Brisbane	19-43	Without CUVAF	17	0	NA	Freehand manual tracing using ImageJ	CUVAF was associated with thinner conjunctival epithelium and thicker conjunctival stroma and sclera.
				With CUVAF	25	Median: 9.2 (Range: 2.5-25.3) Mean (±SD): 10.8 (±6.4)			
Lingham et al., 2023 [32]	Longitudinal	Australia^¥^/Perth	20–28	CUVAF changes with age and sunglass use	Baseline: 1325 1st follow-up: 1073 2nd follow-up: 790	Median CUVAF Baseline visit: 48 (IQR:24.8–72.4), 1st Folow-up: 39.3 (IQR:19.8-61.4), 2nd Follow-up: 37.7 (IQR:18.9 –59.7)	NA	Custom made semi-automated MATLAB analysis tool	CUVAF area declined with age and wearing sunglasses was associated with faster reduction of CUVAF area. The CUVAF area was larger in those who developed pterygium than those that did not.
Bhattacharya et al., 2022 [15]	Cross-sectional	Australia/Brisbane	18–35	Without CUVAF	21	0	Median: 5.6 (6.3) /week	Freehand manual tracing using ImageJ	Individuals with CUVAF had lower corneal epithelial cell density. Suggested that chronic UV exposure damages the corneal microstructure.
				With CUVAF	31	Mean: 9.7 (±7)	Median: 5.3 (6.3)/week		

*BATS* Brisbane Adolescent Twin Study, *G1* group 1, *G2* group 2, *IQR* interquartile range, *NA* not available, *NIES* Norfolk Island Eye Study, *RAINE* The Western Australian Pregnancy Cohort study, *SE* spherical equivalent, *TEST* Twins Eye Study in Tasmania, *UV* ultraviolet, *UVFP* ultraviolet fluorescence photo, and ¥ Raine study cohort, ¢ Norfolk Eye Island study cohort.

**Table 2:** Conjunctival ultraviolet autofluorescence (CUVAF) investigations in Europe.

Authors/Year	Study design	Location	Age (yr)	Population	Number of subjects (*n*)	Mean/Median CUVAF area (mm^2^)	Outdoor exposure time (OET) (h)	CUVAF analysis method	Findings of the study
Wolffsohn et al., 2014 [19]	Cross-sectional	Europe	19–68	Without CUVAF	116	0	NA	Freehand tracing using ImageJ software	The average CUVAF area was high nasally than temporally. CUVAF was not related to age, gender, self-reported outdoor exposure and sunglass use.
				With CUVAF	191	Mean: 5.1 (±4.3)			
Neshkinski et al., 2014 [69]	Prospective randomise	Europe/Varna	18–63	Without CUVAF	200	NA	NA	NA	CUVAF area and intensity increased with age and had negative association with ocular protection.
				With CUVAF					
Kearney et al., 2016 [20]	Cross-sectional	Europe/Ireland	19–64	No dry eye	29	Overall median: 4.9 (IQR: 2.2– 9.4) Overall Mean average pixel intensity (10^3^): 312 (IQR 195– 380)	NA	Custom made MATLAB analysis	CUVAF area and intensity were not associated with clinical measures of dry eye. Larger CUVAF area and intensity was associated with wearing sunglasses less frequently and spending more time outdoors.
				With dry eye	21				
Kearney et al., 2019 [3]	Longitudinal	Europe/Ireland	18–20	Non-myopes	30	Median (IQR): P1: 4.7 (2.2–10.8), P2: 7.5 (2.3–11), P3: 7.7 (1.5–10.2) P4: 7.4 (0.9–10.1) Mean (±SD): P1: 6.4 (±5.4) P2: 7.1 (±5.2) P3: 6.8 (±6) P4: 6.7 (±6.1) Average Pixel intensity (10^3^): Median (IQR): P1: 85 (81–88) P2: 87 (82–92) P3: 93 (86–97) P4: 82 (76–97) Mean (±SD): P1: 72 (±33) P2: 76 (±33) P3: 78 (±36) P4: 73 (±40)	NA	Custom made MATLAB analysis	Myopia was associated with smaller areas of CUVAF indicative of less cumulative ultraviolet-B exposure. These findings suggest that CUVAF measures are a useful, non-invasive biomarker of the time spent outdoors in adults in northern hemisphere populations.
				Myopes SE < −0.50D	24	Median (IQR): P1: 4.9 (0.88–6.7), P2: 4.7 (0.9–5.9), P3: 3.9 (1.2–6.3) P4: 4 (1.5–7.1) Mean (±SD): P1: 6 (±5.7) P2: 5.1 (±4.6) P3: 4.5 (±3.5) P4: 4.2 (±3.7) Average Pixel intensity (10^3^): Median (IQR): P1: 83 (73–86) P2: 85 (81–88) P3: 90 (87–93) P4: 89 (79–94) Mean (±SD): P1: 67 (±36) P2: 75 (±29) P3: 82 (±28) P4: 71 (±39)			
Waszczykowska et al., 2020 [50]	Cross-sectional	Europe/Poland	31–33	Without keratoconus	111	Mean: 7.6 (± 5.1)	NA	Adobe photoshop CC using a standard protocol	The nasal and temporal CUVAF areas were significantly smaller in keratoconus patients compared to the control group. In individuals with keratoconus and primary CUVAF undergoing CXL, a rapid but temporary enlargement of the autofluorescence area was observed.
				With keratoconus	20	Mean: 2.2 (±3.4)			
Ø Bilbao-Malavé et al., 2022 [24]	Cross-sectional	Europe/Spain	Mean 22.4 ( ± 0.8)	Non-myopes	79	Mean: 3.8 (±3.9)	Mean: 12.6 h/week (±10.5)	Freehand manual tracing using ImageJ	The degree of myopia was inversely associated with CUVAF, spherical equivalent refraction and CUVAF were correlated.
				Myopes SE ≤ −1.00D	130	Mean: 2.5 (±2.4)	Mean: 9.4 h/week (±7.4)		
Neshkinski, 2023 [70]	Cross-sectional	Europe/Varna	18–85	Without CUVAF	49	NA	NA	NA	The CUVAF area increased with time and was more commonly seen in eyes with pterygium than without.
				With CUVAF	71				
De La Puente et al., 2024 [40]	Retrospective	Europe/Spain	6–51	Myopes Mild: −0.75 to −3D Moderate: −3.25 to 5.75D High: ≤ −6D	298	NA	NA	NA	CUVAF was more common in children with low myopia compared to high myopia. Suggesting CUVAF as a biomarker of outdoor activity.
de la Puente et al., 2024 [39]	Case–control	Europe/Spain	6–17	Non-myopes	50	Mean: 0.78 (±1.2)	14.3 h/week (±5.7)	Freehand manual tracing using ImageJ	There was a significant difference in CUVAF area in those with and without myopia and CUVAF has an inverse relationship with degree of myopia.
				Myopes mild: −0.75 to -3D moderate: −3.25 to −5.75D High: ≤ -6D	213	Mean: 0.26 (±0.5)	10.6 h/week (±4.5)		

*CXL* Corneal collagen cross-linking, *IQR* interquartile range, *MATLAB* Matrix Laboratory, *P1* Phase1, *P2* Phase2, *P3* Phase3, *P4* Phase4, *ØAge* range not available.

**Table 3:** Conjunctival ultraviolet autofluorescence (CUVAF) investigations in India.

Authors/Year	Study design	Location	Age (yr)	Population	Number of subjects (*n*)	Mean/Median CUVAF area (mm^2^)	Outdoor exposure time (OET) (h)	CUVAF analysis method	Findings of the study
Yadav et al., 2020 [30]	Cross-sectional	India	NA	Without OSSN	17	Mean:15.8 (range: 10.8–19.6)	NA	Freehand manual tracing using ImageJ	CUVAF was noted in the eyes even with negative cytology reports indicating presence of damaged lesion on the ocular surface.
			10–85	With OSSN					
Kumar et al., 2021 [25]	Cross-sectional	India	10–24	Non-myopes	60	Median: 14.4 (IQR:8.4–23.8)	Median: 8 h/week (4.1–11.1)	Freehand manual tracing using ImageJ	There was an inverse relationship between serum melatonin levels and degree of CUVAF in myopes. A novel link between serum melatonin, axial length and outdoor sun exposure was noted.
				Myopes SE ≤ −1.00D	60	Median: 2.8 (IQR:1.2–4.3)	Median: 3.7 h/week (1.8–7.3)		
Ø Pai et al., 2022 [41]	Cross-sectional	India	Mean: 37 ± 10.5	Without pterygium	86	AF was present in 51% of pterygium and most commonly seen in the leading edge (65.9%) of the pterygium.	NA	Freehand manual tracing using ImageJ	AF was mostly seen at the leading edge of pterygium. CUVAF was linked to the severity or grade of pterygium.
			37 ± 13.3	With pterygium	76				
Kumar et al., 2022 [52]	Cross-sectional	India	18–75	Without pinguecula/pterygium	85	Median: 11.1 (IQR: 25.2)	NA	Freehand manual tracing using ImageJ	The nasal conjunctiva had larger areas of CUVAF damage than the temporal area in pterygium patients. Increasing CUVAF was seen with prevalent pterygium.
				With pinguecula/pterygium	144	Median: Pterygium - 45.3 (IQR:35.1) Pinguecula - 17.9 (IQR:16)			
Sureshkumar et al., 2023 [18]	Cross-sectional	India	18–75	Without XFS	274	Median: 4.3 (IQR: 11.2)	NA	Freehand manual tracing using ImageJ	CUVAF was larger in individuals with ocular XFS than those without. CUVAF was greater in those with outdoor work than those with indoor work.
				With XFS	130	Median: 8.8 (IQR: 12.5)			

*AF* autofluorescence, *IQR* interquartile range, *NA* not available, *OSSN* ocular surface squamous neoplasia, *XFS* Pseudoexfoliation syndrome, *ØAge* range not available.

**Table 4:** Conjunctival ultraviolet autofluorescence (CUVAF) investigations in other countries.

Authors/Year	Study design	Location	Age (yrs)	Population	Number of subjects (*n*)	Mean/Median CUVAF area (mm^2^)	Outdoor exposure time (OET) (h)	CUVAF analysis method	Findings of the study
Haworth and Chandler, 2017 [54]	Cross-sectional	USA/	21–64	Without CUVAF	16	0	NA	Freehand manual tracing using ImageJ	Total CUVAF area was higher in winter and with greater outdoor exposure time. Seasonal temperature variations can influence outdoor time.
		Ohio		With CUVAF	34	Median: 6.3 Mean: 9.2 (±9.5)	Mean: 8.9 h/week		
Ø Haworth and Belair, 2020 [21]	Cross-sectional	USA/Ohio	Mean—4.3 ± 2.5	Without CUVAF	15	Mean: 0.6 (±1.2)	NA	Freehand manual tracing using ImageJ	CUVAF was larger in both UV-absorbing and minimally UV-absorbing contact lens wearers, demonstrating contact lens materials may have conjunctival effects.
					29	Mean: Minimally UV-absorbing 3.9 (±6.7) UV-absorbing 4.1 (±7.4)			
				With CUVAF					
Wolffsohn et al., 2022 [71]	Cross-sectional	Multi-centric	18–50	Without CUVAF	105	Mean—3.6 (±3.8)	NA	Freehand manual tracing using ImageJ	CUVAF area did not differ nasally and temporally. CUVAF area was larger in Australian population.
				With CUVAF	105	Mean—3.1 (±3.3)			
Beheshtnejad et al., 2023 [33]	Cross-sectional	Iran	21–73	Without pinguecula/pterygium	25	Mean: 0.4 (±0.5)	NA	Freehand manual tracing using ImageJ	Hypo-autofluorescence patterns occurred in healthy conjunctival AS-AF imaging. A hyper autofluorescence pattern may suggest the early stage of a conjunctival disorder like pinguecula or pterygium.
				With pinguecula/pterygium	50	Mean:Pterygium—7.2 (±4.5) Pinguecula— 19.3 (±7.8)			

*AS-AF* Anterior segment autofluorescence, *IQR* interquartile range, *ØAge* range not available.

Various imaging methods were used to capture autofluorescent areas of the conjunctiva. Thirty studies used a custom-built photographic setup comprising a camera and external UV light source to image CUVAF area. In contrast, three studies used the Heidelberg Retinal Tomography system (Heidelberg-instruments.com) with the Blue-Peak Autofluorescence module (HRA + OCT-BAF) [24, 39, 40], one study used the confocal scanning laser ophthalmoscope [33] and another used a slit-lamp equipped with a cobalt-blue filter to map the CUVAF area [41].

### Qualitative assessment

The quality of included studies was assessed using the NOS tool [35]; individual scores are presented in Supplementary Table 1. Sample size justification was provided in seven studies, and participant selection was described in 29 studies. Comparability of outcomes between participants was reported in 21 studies. Most studies clearly stated their findings and the statistical methods used for analysis were mentioned in 34 studies.

### Geographical variation

**Fig. 2:**
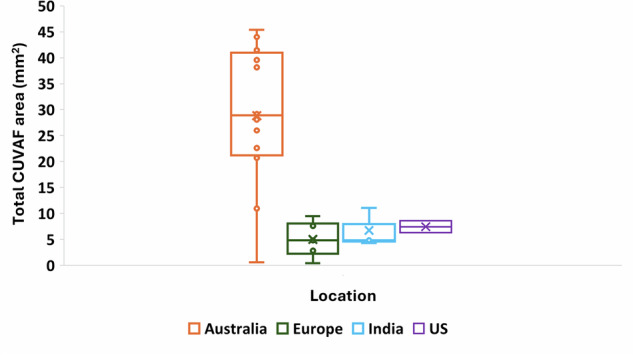
Geographic variation in total conjunctival ultraviolet autofluorescence (CUVAF) area among participants from different studies across various locations (excluding those with ultraviolet (UV)-associated ocular diseases).

Twenty-five studies provided sufficient data to examine the influence of geographic location on CUVAF. In Australian studies (UV index ranging from 2 to 14, [42]) participants exhibited the largest reported CUVAF areas with an overall average of 28.8 mm² [15–17, 26–28, 32, 37, 43–48] (Fig. 2) compared to studies from Europe, India and the USA. The Australian data also demonstrated greater variability (range: 0.6–45.4 mm²) than other geographic regions, which showed relatively narrow CUVAF area measurements clustered between 0.5 and 9 mm².

In contrast, countries with lower ambient UV radiation, such as those in Europe (UVI: 5–9 in summer and 0–1 in winter [49]), reported lower mean/median CUVAF area. The largest reported CUVAF area was 7.6 mm^2^ in healthy eyes without any UV-associated ocular condition [50] and aggregated data from multiple European studies [3, 20, 24, 39, 40, 50] gave an average CUVAF area of 5.3 mm². In India (UVI: 13–10 in summer and 10–3 in winter [51]), the highest reported median CUVAF area was 14.4 mm^2^ in healthy eyes without any UV-associated ocular condition [25], while the average across studies was lower at 6.8 mm² [18, 25, 52]. Similarly, in the USA (mid-western part), (UVI: 3–9 in summer and 0–2 in winter [53]), the average CUVAF area reported across studies was 7.5 mm² [21, 54].

### Age-related patterns

**Fig. 3:**
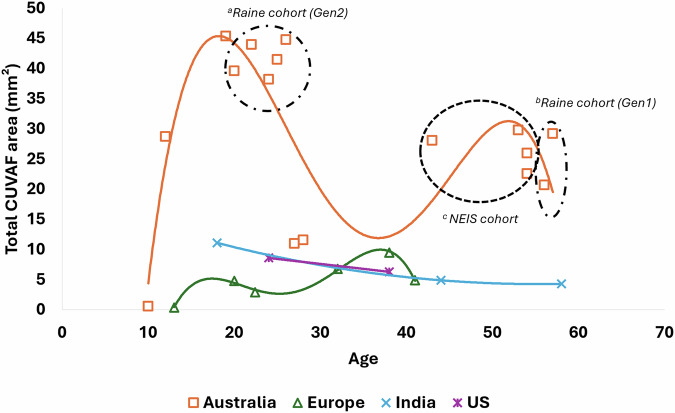
Relationship between age and total conjunctival ultraviolet autofluorescence (CUVAF) area in mm^2^ (weighted CUVAF values depending on the study population e.g., myopic vs. non-myopic; CUVAF present vs. absent), excluding CUVAF area from ultraviolet (UV)-associated ocular diseases). The graph illustrates the non-linear relationship between CUVAF area and age across locations, with polynomial trend lines. Note that age has been plotted as the mean of the study participants and this could span up to 25 years. The data captured were cross-sectional in nature, and the weighted mean and median CUVAF values should be considered as descriptive summaries rather than precise normative estimates, as methodological and population heterogeneity limits the precision of cross-study comparisons. This data may underestimate the true magnitude of age-related variation and between-group differences. ^a^Raine cohort (Gen 1)—The Western Australian pregnancy cohort generation 1 [16, 26, 46, 68]; ^b^Raine cohort (Gen 2)—The Western Australian pregnancy cohort generation 2 [46, 47]*;*^c^*NIES cohort*—Norfolk Island Eye Study [27, 28, 43, 44].

Twenty-five studies reported age-related changes in CUVAF. Based on the CUVAF data (excluding CUVAF area from UV-associated disease) extracted from different studies across various geographic regions, there was a non-linear relationship between CUVAF area and age (Fig. 3). Additionally, LOWESS curve analysis was performed to explore the relationship between CUVAF area and age. This also revealed an irregular curve fit (similar to a polynomial curve), confirming the nonlinear association between CUVAF area and age.

Age-related variations in CUVAF area derived from individuals without UV-associated ocular diseases differed by geographic location, with the caveat that much of the graphed data are cross-sectional rather than longitudinal. In Australian studies, reported CUVAF area increased from 3.8 mm² [17] in childhood to 44.8 mm² [46] in early adulthood, peaking around 20 years of age [16, 32, 45, 46, 48]. This was followed by a dip at age 30 years [15, 37] and a subsequent increase between ages 40 and 60 years [27, 28, 43, 44, 46, 47]. In Europe, reported values ranged from 0.4 mm² [39] to 7.6 mm² [50], with a slight increase in early adulthood [3, 24, 50] followed by oscillation with advancing age [19, 20]. In India, CUVAF decreased from 8.5 mm² in young adults [25] to 4.3 mm² in older individuals [18]. Similarly, in the USA, values showed a slight decrease from 8.6 mm² [21] to 6.3 mm² [54], although the age range studied was limited (~25–40 years).

### Ethnic background and phenotype

Three studies [15, 17, 32], identified ethnic differences in CUVAF distribution, with Caucasians showing larger CUVAF areas than non-Caucasians. Fair skin, lighter eyes and lighter hair colour, along with a history of frequent sunburns and summer freckling, were associated with increased odds of CUVAF [17]. Lingham et al. reported that individuals of East or Southeast Asian ancestry had smaller CUVAF areas (27.9 mm^2^) compared to Caucasians (48.9 mm^2^) and showed minimal changes over time; 26.2–28.6 mm^2^ over an 8-year period [32].

### Seasonal variations

**Fig. 4:**
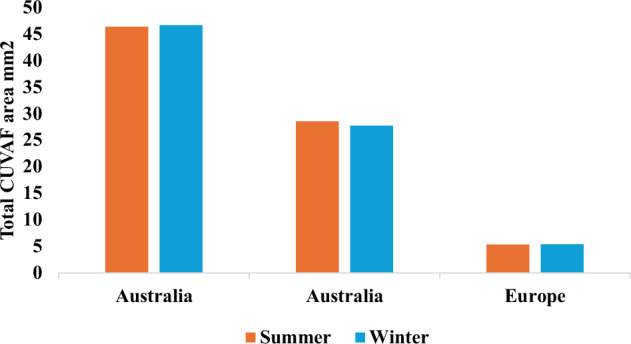
Weighted median conjunctival ultraviolet autofluorescence (CUVAF) area (mm²) of three studies [3, 26, 43] with measures during both summer and winter. There was no observed seasonal variation.

Six studies measured CUVAF area across different seasons, with three providing quantitative data [3, 26, 43] (Fig. 4). In Australia, the Raine Study (*n* = 1344) reported comparable CUVAF areas in summer (46.3 mm²) and winter (46.6 mm²), whereas the Norfolk Island Eye Study (NEIS) cohort (*n* = 49) showed slightly higher values in summer (28.5 mm²) than in winter (27.7 mm²) [26, 43]. In contrast, a European study (*n* = 54) found no seasonal variation, with summer and winter values nearly identical (5.3 mm² versus 5.4 mm²) [3]. Two studies [16, 32] did not include numerical data but noted that CUVAF damage increased with more time spent outdoors in both summer and winter. Additionally, one study [55] reported a rise in CUVAF area in winter (~7.6 mm²) but did not provide spring data.

### Occupational or outdoor exposure

Nine studies examined the relationship between CUVAF area and time spent outdoors; all reported a positive association [15, 16, 20, 25, 28, 32, 39, 44, 54]. Additionally, three studies investigated the link between CUVAF and outdoor occupation [18, 52, 54], with two providing quantitative data. Participants engaged in outdoor occupations had larger total CUVAF areas, averaging 8.5 mm² more than those in indoor roles [54]. Similarly, one study reported CUVAF areas of 7.1 mm^2^ in outdoor workers compared to 3.2 mm^2^ for indoor workers [18].

### Photoprotective behaviours

Data from six published studies found no association with CUVAF area and sunglass use [11, 16, 17, 19, 52, 54]. However, one study reported a negative association, showing that participants who wore sunglasses more than half the time while outdoors had a reduced CUVAF area by an average of −0.42 mm² (95% CI: −0.72 to −0.12) [32]. Similarly, Kearney et al. found that increased frequency of sunglass use was negatively associated with temporal CUVAF area, while nasal CUVAF remained unaffected [20].

### CUVAF in ophthalmohelioses

CUVAF has been reported to be an indicator of UV-related ocular damage in individuals with ophthalmohelioses [41]. The median total CUVAF area was significantly larger in participants with pterygium compared to controls; Raine study [16] (73.4 mm^2^ vs. 44 mm^2^, *p* = 0.001), NEIS [44] (43.4 mm^2^ vs. 26 mm^2^, *p *= <0.001) and in outdoor workers with pterygium [52] (45.3 vs. 11.1 mm^2^, *p* = <0.001). Autofluorescence was also more commonly observed at the limbus and at the leading edge of the pterygium [6].

A significant increase in the likelihood of developing pterygium, ranging from 2.5 to 3.6 times, was observed with increasing quartiles of CUVAF area, suggesting a potential dose-response relationship [16, 44]. CUVAF area was also greater in individuals with pseudoexfoliation syndrome (XFS: 8.8 vs. 4.3 mm² in controls) [18] and in those diagnosed with OSSN (15.8 mm²) [30]. Eyes with CUVAF damage were at an increased risk of developing ocular XFS [18] and OSSN [30].

Across five studies, the average CUVAF area was larger in individuals with UV-related ocular diseases compared to controls (43.4 vs. 20.7 mm^2^) [16, 18, 44, 47, 52]. This finding indicates that elevated levels of ocular UV exposure are typically associated with ophthalmoheliosis [16, 44]. Additionally, eyes with CUVAF showed reduced corneal epithelial cell density, suggesting chronic UV-induced damage to both the corneal microstructure and the conjunctival surface [15].

### CUVAF patterns in the myopic population

Studies investigating myopia have consistently used CUVAF as an objective marker of outdoor activity [3, 25, 26, 28, 48], reporting an inverse relationship between CUVAF area and myopia. Myopes tend to have smaller CUVAF areas than non-myopes [31], a trend observed across multiple regions. In Australia, CUVAF areas were 47.9 mm² for non-myopes and 31.9 mm² for myopes [26]. In India, respective values were 14.4 versus 2.8 mm² [25], compared with 3.8 versus 2.5 mm² in Spain. [24]. Data from eight studies support this pattern, with non-myopes having a higher average CUVAF area of 21.5 mm² compared to 14.5 mm² in myopes [3, 24–26, 28, 39, 46, 48]. However, most studies did not specify the season of data collection or the timeframe over which UV-related damage developed.

### Acute artificial ultraviolet exposure

Eyes exposed to acute UVA irradiation during corneal collagen cross-linking (CXL) for the treatment of keratoconus showed a temporary increase in CUVAF area. Measurements indicated an increase from 2.2 mm² before CXL, to 8.4 mm² at 7 days post-treatment, followed by a decrease to 6.5 mm² after 30 days. These findings suggest that short-term, concentrated UV exposure can cause cellular injury and induce tissue autofluorescence within a brief period [50].

## Discussion

CUVAF appears to be a reliable marker of past UV exposure; however, its presence, size and intensity are influenced by multiple factors, and it is generally assumed to reflect past cumulative sun exposure seen after several months. Emerging evidence also suggests that CUVAF may indicate acute cellular responses or ongoing tissue injury following short-term UV exposure, highlighting its potential utility in detecting both chronic and recent UV-induced ocular damage.

### Regional distribution

CUVAF area varied with geographical location, with larger areas observed in the Australian studies, likely due to Australia’s high ambient UV index, averaging 10–14 in summer and 2–7 in winter, depending on latitude [42]. Repeated UV exposure causes ocular surface damage, leading to increased oxidative stress and early tissue damage that presents as autofluorescence [56]. This is consistent with Australia’s high skin cancer rates (127.6 cases/100,000 in 2021), compared to 27–55.6 cases in Europe and 3.7 cases in South Asia. Despite North America’s high overall skin cancer incidence (730.2 cases/100,000), the CUVAF area was lower [57]. These regional differences in CUVAF may be influenced by factors beyond the UV Index, such as outdoor lifestyles, skin type, proximity to the equator and environmental factors such as ozone depletion [58].

Within the Australian population, CUVAF varied considerably. The NIES study reported a median CUVAF of 28.2 mm² [27], while the Raine study noted a value of 44.2 mm² [16]. These differences could be attributed to latitude, weather patterns, UV exposure behaviours [28], age-related factors, outdoor activity levels or group/period effects [27]. Additionally, McKnight et al. (Raine) [26] and Sherwin et al. (NEIS) [44] found that individuals spending none or <1/4 time outdoors still had a CUVAF area of 24.8 and 21.8 mm², respectively, possibly due to past sun exposure, extreme UV events or inadequate ocular protection. Despite being a comparable high-UV region of Australia, studies conducted in Brisbane reported lower mean/median CUVAF areas of 9.7 mm^2^ [15] and 9.2 mm^2^ [37] compared with those observed in the Raine and NIES cohorts [16, 44]. This discrepancy may be attributed to population differences; the NIES cohort comprised 100% Caucasian long-term residents [44], while the Raine cohort included 85.5% Caucasian participants [16]. In contrast, the Brisbane studies reported a more ethnically diverse population, with 40.4% [37] to 92.3% [15] of participants identifying with non-Caucasian backgrounds.

This pattern aligns with Indian data, showing lower CUVAF (4.3–11.1 mm²) [18, 25, 52] despite high UV exposure, likely due to higher melanin content providing photoprotection [59]. The conjunctiva contains resident dendritic melanocytes in the basal layer of the epithelium, presenting as flat brown pigmentation known as primary conjunctival hyper-melanosis (complex related melanosis), which is more prevalent in darker skin types and Asians [59]. These melanocytes have elongated cell processes called dendrites that transfer melanin granules into conjunctival epithelial cells, absorbing harmful UV radiation to protect the conjunctiva from damage [60].

Comparisons of CUVAF across ethnic groups were not adjusted for confounders such as migration, residential history, occupational exposure patterns, socioeconomic status and behavioural sun protection practices. As a result, the observed ethnic differences in CUVAF cannot be attributed solely to geographic location. Behavioural and environmental factors that differ systematically between ethnic groups are likely to contribute substantially to the reported variations in CUVAF area. Therefore, current evidence supports only a cautious interpretation of ethnic differences in CUVAF, and well-designed longitudinal studies with detailed adjustment for UV exposure patterns and protective behaviours are required to distinguish biological pigmentation effects from lifestyle and environmental influences.

### Demographic characteristics

Although a non-linear relationship between age and CUVAF area has been reported across studies (Fig. 3), the apparent age-related patterns must be interpreted cautiously because most available data are cross-sectional. Consequently, the observed fluctuations cannot be assumed to represent true within-individual changes across the lifespan and may also reflect cohort effects, differences in sample composition, generational variation in outdoor exposure and sun-protection behaviours or methodological artefacts related to study-level aggregation. While biological repair mechanisms and dynamic cellular responses to UV exposure remain plausible, there is currently insufficient longitudinal evidence to support such interpretations. Definitive characterisation of CUVAF trajectories, therefore, requires prospective studies with repeated measurements and concurrent objective assessment of UV exposure.

A frequently reported finding is that CUVAF peaks in early adulthood [16, 26], a pattern hypothesised to reflect higher outdoor activity and reduced ocular protection during this life stage [32]. In Australian cross-sectional studies, CUVAF increases from childhood to young adulthood, followed by greater variability in later life, whereas European data demonstrate only a modest early-adult rise with a similar oscillating pattern, thereafter, possibly reflecting behavioural change or improved sun-protection practices with age [32, 61]. In contrast, Lingham et al. [32] reported a longitudinal decline in median CUVAF area from 48.0 mm² at baseline to 39.3 and 37.7 mm² at successive follow-ups, corresponding to an average annual reduction of ~0.96 mm². Importantly, participants who consistently wore sunglasses for at least half of their outdoor exposure exhibited a significantly faster decline in CUVAF, highlighting the modifying role of behavioural photoprotection [32]. Active tissue repair mechanisms have also been proposed as one of the contributing factors to CUVAF reduction [3, 56]. However, more longitudinal studies are required to confirm this proposal. Findings from India and the USA remain difficult to interpret due to the limited number of available studies.

Therefore, CUVAF expression appears to be influenced by multiple factors, such as UV exposure, geography, lifestyle and behavioural factors. Higher CUVAF values in younger individuals suggest that CUVAF may reflect moderately accumulated ocular UV exposure. However, this interpretation remains speculative due to the lack of longitudinal evidence. Age-related trends may also result from group effects or sample composition [16]. Additionally, using weighted mean or median values in the graph can cause data points to cluster around aggregate population trends, as seen in Australian studies. This aggregation effect may underestimate true differences between groups.

### Seasonal dynamics

The majority of studies found no seasonal variation in CUVAF area [3, 16, 17, 26, 43]. However, one investigation [54] reported larger CUVAF damage during the winter compared with the spring. This finding may reflect increased outdoor activity during the winter months or the conjunctival tissue’s capacity to retain autofluorescent properties following prior UV exposure.

The persistence of autofluorescence may be attributed to cellular-level changes following UV exposure. Alterations in intracellular protein content, particularly in endogenous components, such as lysosomes, mitochondria, cytokines, growth factors and MMPs, can remain within conjunctival cells long after the initial exposure [24]. This suggests that CUVAF signals detected during winter may represent residual effects from previous periods of high UV exposure. However, the absence of seasonal differences in most studies may be due to data collection occurring at fixed time points, potentially missing intra-seasonal fluctuations. Additionally, individual variations in sun exposure patterns may have masked seasonal trends.

### CUVAF changes in myopes

Evidence linking CUVAF and myopia supports the association between time spent outdoors and CUVAF area. The observation of smaller CUVAF areas in individuals with myopia suggest reduced outdoor activity in this refractive group [3, 25, 26, 28]. A meta-analysis found that myopes (*n* = 3615, age range 10–89 years) had 3.3 mm² less CUVAF and self-reported 3.4 fewer hours outdoors per week [31]. These associations have been interpreted as supporting the hypothesis that outdoor time protects against myopia development [31].

Outdoor light exposure is thought to stimulate dopamine release in the retina, which helps slow axial elongation and reduces the risk of myopia [62]. However, factors such as socioeconomic status, near-work habits and parental myopia can influence both time spent outdoors (and thus CUVAF area) and myopia risk [62]. While CUVAF primarily reflects cumulative UV exposure to the conjunctiva, the protective effect against myopia is believed to be mediated primarily by visible light. Therefore, a smaller CUVAF area likely indicates less overall outdoor light exposure. This explains why the observed link between smaller CUVAF and myopia is consistent with reduced time outdoors and lower exposure to both visible and UV light.

### Pathological associations of CUVAF

Early-life UV exposure may increase the risk of developing UV-related ocular diseases later in life [7]. CUVAF area was significantly greater in individuals with ophthalmohelioses compared to healthy eyes. The dose–response relationship identified between CUVAF quartiles and pterygium development suggests it as a quantifiable threshold effect where long-term UV damage progressively increases disease risk [16, 44]. This finding aligns with established models of UV-induced carcinogenesis and supports the hypothesis that CUVAF accumulation represents a gradual, dose-dependent process of cellular damage.

The association of CUVAF with ophthalmohelioses [16, 18, 41, 44] indicates a shared pathophysiological mechanism mediated by chronic UV exposure. Damage at the limbus, as well as the observed reduction in corneal epithelial cell density [15] and altered conjunctival epithelium [37] suggest that CUVAF reflects both surface and deeper microstructural damage, with injury to the ocular surface microstructures, which may contribute to progressive deterioration of ocular health over time [15].

### Acute UV effects

Exposure to high-dose therapeutic UVA during CXL for keratoconus causes a temporary increase in CUVAF area. This effect is unlikely under normal environmental UV conditions. However, prolonged high-intensity daily exposure, such as during outdoor activities in snow or beach settings without eye protection, can lead to photodamage of the ocular surface [56]. These findings suggest that intense, concentrated UV exposure can produce detectable CUVAF within days, most likely through oxidative stress and inflammatory processes [56, 63].

Consistently, greater time spent outdoors has been linked with larger CUVAF areas. This association is attributed to UV-induced activation of pro-inflammatory molecules, including interleukins, cytokines and MMPs, which contribute to cellular injury [50]. Both ambient and artificial UV exposure induce oxidative stress on the ocular surface, leading to molecular alterations in tissue structures [4]. These changes damage proteins, lipids and DNA [56]. In the conjunctiva, UV radiation can elevate inflammatory mediators such as IL-1β, IL-6, TNF-α, IL-17 and IFN-γ [63], and this localised inflammation may contribute to DNA damage and the development of active autofluorescence [19].

### Modelling limitations

LOWESS analysis effectively addressed the skewed distribution of CUVAF area across age groups. The resulting exploratory graph revealed a non-linear relationship between age and CUVAF area. The curve was generated using a medium smoothing span, with 95% confidence intervals. Additional analyses with finer spans produced similar patterns, confirming that the observed oscillations were not artefacts of over- or under-smoothing. Larger CUVAF values appeared in younger individuals, followed by a decline during middle adulthood and a subsequent increase in older age groups. To evaluate the robustness of this pattern, a polynomial model was also fitted. Both modelling approaches reproduced the same inflection points, indicating that the fluctuations across the lifespan reflect underlying data structure rather than modelling artefacts.

Additionally, more sophisticated modelling approaches, such as GAMs, could be used to examine associations involving nonlinear data and their predictors; however, these approaches require individual-level data. In the current study, only group-level summary statistics (e.g., means or medians) were available, which are not suitable for GAM fitting because they may obscure the underlying data distributions. Therefore, polynomial and LOWESS plots were used solely for data visualisation purposes.

This trend may be influenced by behavioural shifts, occupational patterns or increased use of sun protection measures [47, 64]. In older age groups, the CUVAF area increased again, indicating renewed cumulative photodamage. These fluctuations across the lifespan imply that CUVAF reflects moderately cumulative damage rather than short-term exposure. While polynomial and LOWESS smoothing are useful for visualising complex patterns, each has limitations. These limitations highlight the exploratory nature of the analyses and underscore the need for complementary modelling approaches when interpreting non-linear relationships.

### CUVAF repeatability

The methodological robustness of CUVAF as a measurement tool has been evaluated across multiple studies. Lingham et al. [65] reported high repeatability, with intra- and inter-observer correlations exceeding 0.9, while Huynh et al. [66] demonstrated 96% inter- and intra-observer agreement for a semiautomated analysis, with a narrow limit of agreement (LOA) despite modest mean differences compared to manual assessments.

Haworth et al. [54] supported these findings, reporting near-zero mean differences and narrow LOA in test-retest evaluations, with no systematic bias. These results were consistent with earlier validation work by Sherwin et al. [43] in a genetically isolated cohort. Lingham et al. [32] evaluated a deep learning-based CUVAF segmentation tool, reporting minimal mean differences between automated and semiautomated methods, excellent intra-examiner repeatability and significantly reduced examination time.

Collectively, these studies demonstrate strong repeatability and minimal observer-dependent variability across manual, semiautomated and deep learning-enhanced approaches. Although CUVAF demonstrates strong reliability within individual studies, it is hard to extrapolate this more broadly across studies due to large variations in instrument setup and analysis protocols across research groups.

### CUVAF methodological limitations

Although CUVAF measurements generally show high reliability, variability across studies is likely driven by methodological differences. Most studies use similar camera systems with external UV light sources (300–400 nm), but image analysis methods vary widely, including semi-automated approaches using Photoshop (adobe.com), ImageJ (imagej.net) and MATLAB (mathworks.com). The absence of a standardised grading system further limits comparability. Differences in operator input, analytical workflows and incomplete reporting of camera parameters (such as camera-to-eye distance, International Organisation for Standardisation (ISO) sensitivity and shutter speed) may have contributed to calibration differences and inter-study variation.

Study populations also differed, spanning Australia, India, the USA and Europe, with wide variation in age, pigmentation, occupational and recreational sun exposure, geographic residence and sun-protection behaviours. Behavioural exposure measures were inconsistent; time spent outdoors was often self-reported using questionnaires with varying recall periods and sun-protection behaviours were defined using heterogeneous criteria. Similarly, iris colour and skin pigmentation were classified using different systems, from broad categories to detailed scales such as the Fitzpatrick phototype [67], limiting standardisation of key confounders. As most data were cross-sectional, weighted mean, and median CUVAF values in graphs should be interpreted as descriptive summaries rather than normative reference values.

Finally, CUVAF represents a dynamic biomarker influenced by multiple environmental and biological factors, and single-time-point measurements may not capture its developmental trajectory. In myopia research, CUVAF reflects UV-induced conjunctival changes and should not be considered a direct proxy for total outdoor light exposure. Non-linear age-related patterns, explored using polynomial regression and LOWESS smoothing, remain exploratory and sensitive to model specification. These limitations underscore the need for longitudinal studies and complementary modelling strategies to better characterise age-related changes in CUVAF.

### Future directions

Despite its potential as a biomarker of outdoor ocular UV exposure, CUVAF requires standardised imaging protocols and analytical methods to support broader clinical applications, particularly for early detection of UV-induced damage. Longitudinal studies with repeated measurements and concurrent UV dosimetry are essential to establish CUVAF’s temporal dynamics and how it responds to changes in UV exposure over time. This is necessary to validate its application as a cumulative UV exposure biomarker, a dynamic indicator of recent UV exposure or a marker with both applications. CUVAF holds promise as a population-level screening tool for quantifying the ocular damage in individuals at risk of UV-related eye conditions.

## Conclusion

Cross-sectional evidence shows that CUVAF area varies systematically with factors linked to UV exposure. Regions with higher ambient UV report larger CUVAF, outdoor workers have greater CUVAF than indoor workers and fair-skinned populations have higher CUVAF compared with darker-pigmented groups. These patterns support the use of CUVAF as a biomarker of UV exposure history. Experimental evidence from CXL demonstrates that intense artificial UVA irradiation can produce measurable increases in CUVAF within days, demonstrating rapid conjunctival autofluorescence under extreme conditions. However, whether everyday environmental UV exposure produces similarly dynamic changes remains unknown due to the lack of longitudinal studies tracking CUVAF alongside measured ambient UV exposure.

## Supplementary information

Supplementary information
