# Supplementary material for: Conjunctival Ultraviolet Autofluorescence: A Systematic Review of Factors Affecting Observed Ocular Damage
Source: Ophthalmic Physiol Opt. 2026 Feb 27;46(2):305–21. doi: 10.1007/s44402-026-00043-1 (PMC13369736; doi:10.1007/s44402-026-00043-1)
Supplement: Supplementary file 1 — Supplementary information [file 44402_2026_43_MOESM1_ESM.docx]

Supplementary table 1. Quality assessment of included studies using the Newcastle-Ottawa Scale tool

| Publication: Authors., Year | Selection | | | | Comparability | Outcome | | Quality score |
| --- | --- | --- | --- | --- | --- | --- | --- | --- |
|  |  |  |  |  |  |  |  |  |
|  |  |  |  |  |  |  |  |  |
|  | Representativeness of sample | Sample size | Non-response rate | Ascertainment of exposure |  | Assessment of outcome | Statistical tool |  |
| Bilbao-Malavé et al., 2022 |  |  | * | ** | * | * | * | 6 |
| Bhattacharya et al., 2022 | * |  | * | ** | * | * | * | 7 |
| Beheshtnejad et al., 2023 |  |  |  | ** |  |  | * | 3 |
| Charng et al., 2019 | * |  | * | ** | * |  | * | 6 |
| De La Puente et al., 2024b | * |  | * | ** | * | * | * | 7 |
| de la Puente et al., 2024a | * |  |  | ** | * | * | * | 6 |
| Haworth and Chandler, 2017 | * |  |  | ** | * | * | * | 6 |
| Haworth and Belair, 2020 |  |  |  | ** | * |  | * | 4 |
| Kearney et al., 2016 |  | * | * | ** | * | * | * | 7 |
| Kearney et al., 2019 |  | * | * | ** | * | * | * | 7 |
| Kumar et al., 2021 | * | * | * | ** | * | ** | * | 9 |
| Kumar et al., 2022 | * |  | * | ** |  | * | * | 6 |
| Lingham et al., 2021 | * |  | * | ** |  | ** | * | 7 |
| Lee et al., 2022 | * |  | * | ** |  | * | * | 6 |
| Lingham et al., 2023 | * |  |  | ** | ** | * | * | 7 |
| McKnight et al., 2014 | * |  |  | ** | * | * | * | 6 |
| McKnight et al., 2015 | * |  | * | ** | * | * | * | 7 |
| Neshkinski et al., 2014 | * |  | * | ** |  |  | * | 5 |
| Neshkinski, 2023 | * |  |  | ** |  |  | * | 4 |
| Ooi et al., 2006 | * |  |  | ** |  |  | * | 4 |
| Ooi et al., 2007 |  |  |  | ** |  |  |  | 2 |
| Rajasingam et al., 2023 | * |  | * | ** | ** | * | * | 8 |
| SV et al., 2022 | * |  | * |  |  | ** | * | 5 |
| Sherwin et al., 2011 | * |  | * | ** | * |  | * | 6 |
| Sherwin et al., 2012a | * |  | * | ** | ** | * | * | 8 |
| Sherwin et al., 2012b | * |  | * | ** | * | * | * | 7 |
| Sherwin et al., 2013 | * | * |  | ** | * | * | * | 7 |
| Stevenson et al., 2021 | * |  | * | ** | * | * | * | 7 |
| Sun et al., 2017 | * | * | * | ** |  | * | * | 7 |
| Sureshkumar et al., 2023 | * |  | * | ** | * | * | * | 7 |
| Wolffsohn et al., 2014 | * |  |  | ** | ** | * | * | 7 |
| Waszczykowska et al., 2020 | * | * | * | ** |  |  | * | 6 |
| Wolffsohn et al., 2022 | * | * | * | ** |  | * | * | 7 |
| Yazar et al., 2015 | * |  | * | ** |  | * | * | 6 |
| Yadav et al., 2020 | * |  |  | ** |  | ** | * | 6 |
